# T cells with low CD2 levels express reduced restriction factors and are preferentially infected in therapy naïve chronic HIV-1 patients

**DOI:** 10.7448/IAS.20.1.21865

**Published:** 2017-09-19

**Authors:** Sebastian Bolduan, Herwig Koppensteiner, Ramona Businger, Stephanie Rebensburg, Christine Kunze, Ruth Brack-Werner, Rika Draenert, Michael Schindler

**Affiliations:** ^a^ Institute of Virology, Helmholtz Zentrum Munich, Munich, Germany; ^b^ Institute of Medical Virology, University Hospital Tuebingen, Tübingen, Germany; ^c^ Medizinische Klinik und Poliklinik IV, Section Clinical Infectious Diseases, Klinikum der Universität, Munich, Germany

**Keywords:** HIV-1, restriction factors, *in vivo* relevance, SAMHD1, p21, RISP, Tetherin, SerinC5

## Abstract

**Introduction**: Restriction factors (RFs) suppress HIV-1 in cell lines and primary cell models. Hence, RFs might be attractive targets for novel antiviral strategies, but their importance for virus control *in vivo* is controversial.

**Methods**: We profiled the expression of RFs in primary blood-derived mononuclear cells (PBMC) from therapy-naïve HIV-1 patients and quantified infection.

**Results**: Overall, there was no correlation between individual RF expression and HIV-1 status in total PBMC. However, we identified a T cell population with low levels of intracellular CD2 and reduced expression of SAMHD1, p21 and SerinC5. CD2^low^ T cells with reduced RF expression were markedly positive for HIV-1 p24. In contrast, CD2+ T cells were less infected and expressed higher levels of RFs. CD2^low^ T cell infection correlated with viral loads and was associated with HIV-1 disease progression.

**Conclusions**: In untreated therapy naïve chronic HIV-1 patients, RF expression in T cells is associated with CD2 expression and seems to influence viral loads. Our study suggests that RFs help to control HIV-1 infection in certain T cells *in vivo* and supports the potential for RFs as promising targets for therapeutic intervention.

## Introduction

Various cellular gene products interfere with virus replication. These cellular factors are termed virus restriction factors (RFs) [1]. The function of RFs in cellular processes are in many cases unclear and it is tempting to speculate that they are in first-order components of the so-called host intrinsic immunity for frontline protection against virus infections [1].

In general, RFs are capable of significantly decreasing production of infectious virus and many viruses developed strategies to antagonize the antiviral activity of RFs. Moreover, expression of many RFs is inducible by interferons and RFs often show signatures of rapid evolution by positive selection of conserved amino acid residues [1,2]. In the last years, a series of RFs attacking HIV at various stages of the viral replication cycle were identified [2,3]. Targeting the activity of RFs in the context of antiviral therapy or vaccination seems an attractive approach. However, up to now, clear evidence for the *in vivo* importance of RFs for HIV-1 control is largely missing, controversial [4–7] and/or is derived from non-human *in vivo* models [8–13].

We initiated this study to analyse the importance of RFs in antiretroviral treatment naïve HIV-1 patients that control the infection or, alternatively, progressed to high viral loads. In patient-isolated peripheral blood mononuclear cells (PBMC), we profiled transcription and protein expression of four RFs that inhibit HIV-1 at different stages of viral replication. RFs investigated include the sterile alpha motif (SAM) and histidine-aspartate (HD) domain-containing protein 1 (SAMHD1), which inhibits reverse transcription of the viral genome by lowering the dNTP pool [3,14,15]. The cyclin-dependent kinase (CDK) inhibitor p21 (also termed Waf1/Cip1) interferes with HIV-1 integration and restricts early replication in CD4+ T cells, macrophages and hematopoietic cell lineages [16–18]. The Rev interacting protein (RISP) restricts HIV-1 production in astrocytes by inhibition of HIV-1 Rev [19]. Tetherin inhibits HIV-1 release from sites of viral budding and assembly at the plasma membrane [20,21]. Furthermore, we analysed a smaller subset of patients for the recently identified suppressor of HIV-1 infectivity SerinC5 [22,23]. We observed no general differences in RF expression in total PBMC between patients with disease progression or patients controlling the infection. However, we identified a CD4+ T cell population with low levels of intracellular CD2 and high HIV-1 infection rates in comparison to CD4+ CD2+ cells. Strikingly, CD4+ CD2^low^ T cells expressed reduced levels of RFs SAMHD1 and p21 and HIV-1 p24 staining in these cells was associated with viral loads. Overall, our results indicate that RF expression could influence infection rates in HIV-1 patients and could hence be determinants of HIV-1 control *in vivo.*

## Methods

### Ethic approval and consent to participate

44 individuals participated in the study after signing informed consent. The study was approved by the IRB of the Ludwig-Maximilians-Universität, Munich, Germany (Project No. 274-03). All experiments were performed in compliance with relevant laws and institutional guidelines and in accordance with the ethical standards of the Declaration of Helsinki.

### Study subjects

All study subjects were ART naïve and divided into the following groups [1]: HIV-1 viraemic controllers, defined by CD4 counts >400 cells/µl and viral loads <5000 copies/ml and [2] HIV-1 progressors with CD4 counts <400 cells/µl and viral loads >10,000 copies/ml (details see Table 1).

**Table 1.** Characteristics of HIV-1-positive individuals included in this study.

Controllers					Progressors				
ID	CD4 T cells	Viral load	Gender	Age	ID	CD4 T cells	Viral load	Gender	Age
CO 01	765	201	m	53	PR 01	293	181,497	m	38
CO 02	477	498	f	32	PR 02	204	69,915	m	33
CO 03	782	3101	f	43	PR 03	150	<500,000	m	34
CO 04	517	2247	f	51	PR 04	221	151,857	m	34
CO 05	473	2835	m	58	PR 05	395	152,867	m	62
CO 06	715	3286	m	43	PR 06	297	11,683	m	53
CO 07	547	103	f	32	PR 07	58	11,805	m	46
CO 08	1578	682	m	39	PR 08	362	49,129	m	35
CO 09	413	3032	m	67	PR 09	372	36,824	m	37
CO 10	653	2567	m	50	PR 10	251	68,277	m	73
CO 11	456	472	m	39	PR 11	277	131,229	m	32
CO 12	655	1739	m	26	PR 12	207	44,023	m	42
CO 13	670	<50	f	48	PR 13	190	43,551	m	48
CO 14	500	3421	m	31	PR 14	326	113,164	m	44
CO 15	586	933	m	54	PR 15	193	43,021	m	56
CO 16	1050	1978	f	30	PR 16	246	56,407	m	33
CO 17	1532	<50	m	62	PR 17	196	32,523	m	43
CO 18	833	2142	f	73	PR 18	227	58,272	f	60
CO 19	807	865	m	47	PR 19	341	62,625	m	39
CO 20	598	2180	f	25	PR 20	268	66,885	f	29
CO 21	529	386	m	24	PR 21	290	20,576	m	30
CO 22	785	3739	f	34	PR 22	205	18,000	m	34

**CD4 T cell counts are measured as the absolute number of peripheral T cells per μl blood. Similarly, viral load is the absolute number of proviral copies per ml serum. Controllers are defined by viral loads less than 5000 copies/ml and CD4 T cell counts higher than 400 cells/μl. In contrast, progressors have viral loads higher than 10,000 copies/ml and CD4 T cell counts less than 400 cells/μl. Controllers have a stable course of infection for at least six years. All patients are antiretroviral treatment naïve or have not received antiretroviral therapy for at least 2** **years.**

From each patient, we had one aliquot of PBMC available that were isolated from approximately 10 ml EDTA blood by Ficoll density-gradient centrifugation and stored in liquid nitrogen until further usage. We also isolated PBMC from three individuals on antiretroviral therapy with no detectable viral loads and high and stable CD4+ T cell counts. For analyses of healthy donor RF expression in PBMC and *ex vivo* infection experiments, we isolated PBMC from buffy coat as described [24].

### RNA isolation and qRT-PCR

RNA was isolated from PBMC using the RNeasy Mini Kit (Qiagen) according to the manufacturer’s protocol. RF mRNA levels of Tetherin, SAMHD1, p21 and RISP were determined by One-Step qRT-PCR Kit (Roche) according to the manufacturer’s protocol using specific primer pairs for amplification of *Tetherin* (forward primer: 5´-CTGCAACCACACTGTGATG-3´; reverse primer: 5´-ACGCGTCCTGAAGCTTATG-3´) [25], *SAMHD1* (forward primer: 5´-TCGTCCGAATCATTGATACACC-3´; reverse primer: 5´-CCAGTGCGTGAACTAGACATCC-3´) [26], p21 (forward primer: 5´-GGAAGACCATGTGGACCTGT-3´; reverse primer: 5´-GGCGTTTGGAGTGGTAGAAA-3´) [27], *RISP* (forward primer: 5´GGAAGCAATTAAACCCTCTCA-3´; reverse primer: 5´-TTTGGTTTTACAGTTAAGTCAGCAA-3´) and *RNA polymerase II* (forward primer: 5´GCACCACGTCCAATGACAT-3; reverse primer: 5´-GTGCGGCTGCTTCCATAA-3´) [28]. Quantitative RT-PCR was performed as described [29].

### Flow cytometry analysis of RF expression

We aimed to measure intracellular CD2, HIV-1 p24 and the expression of a RF in one staining step. Hence, for the intracellular stainings, PBMC were fixed with 2% PFA for 20 min at 4°C and permeabilized with 1% Saponin for 10 min at RT. Afterwards, cells were separated into different aliquots and intracellularly stained with one of the following antibodies: either human Tetherin-Alexa Fluor 647 (BioLegend), p21 (Proteintech), SAMHD1 (Proteintech), RISP [19] or SerinC5 (Abcam). Then cells were stained with the corresponding secondary antibodies either anti-mouse, anti-rat or anti-rabbit Alexa Fluor 633 (Molecular Probes, Invitrogen). In addition, we used anti-p24-FITC (Beckman Coulter Clone KC57) and anti-CD2-Pacific blue (BioLegend).

For the surface staining we used either anti-CD3-APC (Invitrogen), anti-CD4-APC (Invitrogen), anti-CD62L-APC (BioLegend) or Tetherin-Alexa Fluor 647 (BioLegend). Then, cells were intracellularly stained for p24 and CD2 as described above. We measured with a FACS CANTOII with similar PMT voltage settings throughout the study and analysed the data with the FACSDiva software (BD Biosciences) or Flowlogic (Miltenyi/Inivai).

### *Ex vivo* HIV-1 infection of PBMC

PBMC isolation from buffy coat, stimulation, production of HIV-1 NL4-3 virus stock and quantification of p24 was done as described [24]. 48 h post infection cells were harvested and stained for surface CD3 (anti-CD3-VioBlue, Miltenyi) and intracellular HIV-1 with anti-p24-FITC as described above. CD3 expression levels and HIV-1 infection was analysed by flow cytometry.

### Statistical analyses

We used GraphPad Prism 6.0 for all statistical calculations. Data sets were first assessed for normal distribution with the D’Agostino-Pearson omnibus test. Then, depending on normal- or non-normal data distribution we tested for significance with the appropriate test as indicated in the figure legend of each data set.

## Results

### Profiling of RF transcript levels in PBMC from HIV-1 controllers and progressors

The expression of RFs could be an important determinant of HIV-1 disease progression in patients. We therefore measured the expression levels of the RFs SAMHD1, p21, RISP and Tetherin at mRNA level in a cohort of therapy naïve HIV-positive individuals.

The baseline characteristics of HIV-1 patients analysed in this study are outlined in Table 1. CD4+ T cell counts inversely correlated with viral loads (Spearman *r* = −0.7439; *p* < 0.0001) whereas patient age was neither associated with viral loads nor CD4+ T cell counts.

**Figure 1.**
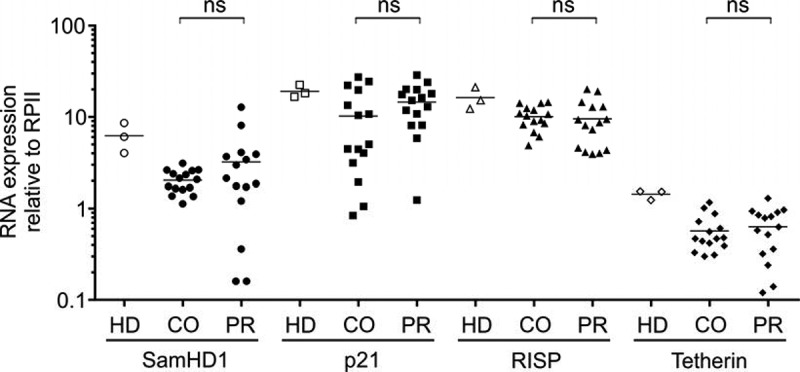
*Quantification of RF mRNA levels in PBMC of HIV-1-positive controllers* versus *progressors*. RF mRNA levels of SAMHD1, p21, RISP and Tetherin in HIV-1-positive controllers (CO) and progressors (PR) of 15 donors were determined by One-Step qRT-PCR Kit (Roche) using specific primer pairs (see Material and Methods). The mRNA expression was plotted relative to the mRNA levels of the *RNA polymerase II* (RPII). Each data point represents the specific mRNA expression of the indicated RF in one patient. For comparison to RF mRNA levels in uninfected individuals, we also profiled PBMC from three healthy donors (HD). Mean values of RF mRNA expression of controllers and progressors were analysed for statistical differences using an unpaired two-tailed Mann–Whitney test assuming non-Gaussian distribution (Graph Pad Prism 6). ns, not significant.

Total RNA was isolated from patients’ PBMC and RF expression was determined in 15 viraemic controllers and 15 progressors by qRT-PCR. The RNA expression pattern of the various RFs was divergent, however, in a similar range as the RNA levels in PBMC directly isolated from buffy coats of healthy individuals (Figure 1).

PBMC mRNA levels of SAMHD1, p21, RISP and Tetherin did not show any significant differences between HIV-1-positive individuals controlling the infection or conversely showing a progressive course of infection. Closer examination of any potential association between RF mRNA expression and CD4+ T cell counts or viral load also did not reveal any significant correlation (data not shown). In conclusion, mRNA levels of SAMHD1, p21, RISP or Tetherin in PBMC of HIV-1 patients do not show a specific association with HIV-1 control or viral loads.

### Profiling of RF protein expression in PBMC of HIV-1-positive viraemic controllers and progressors by flow cytometry

**Figure 2.**
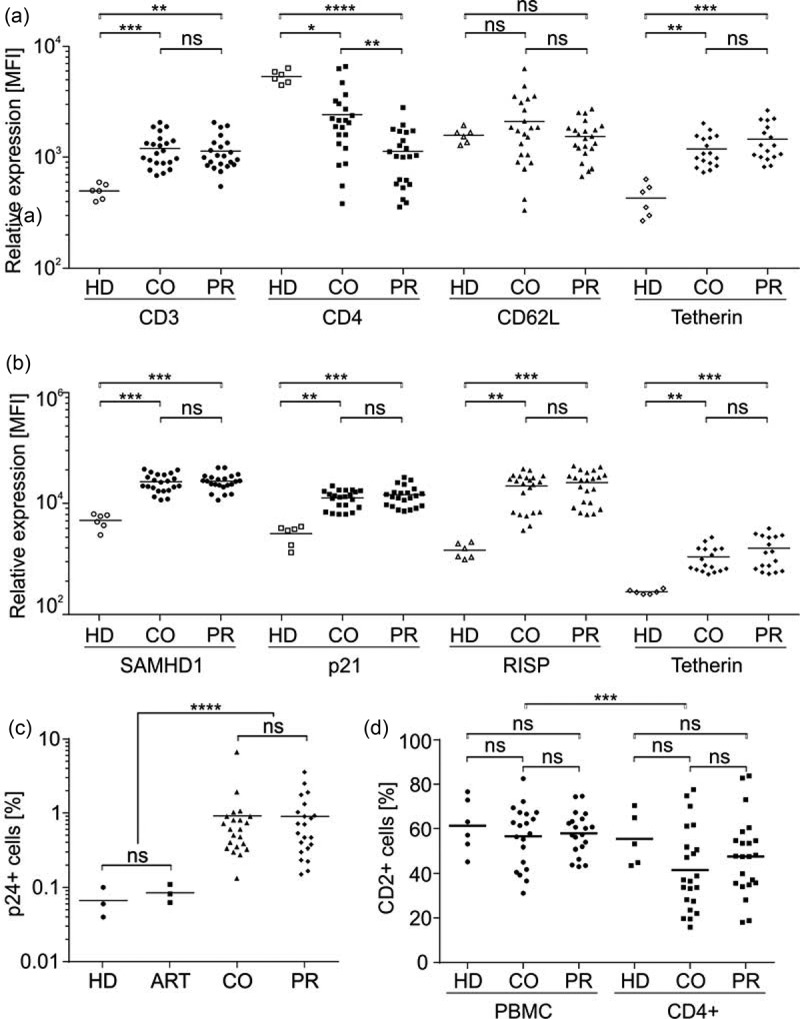
*Quantification of host cell factors, intracellular p24 as well as CD2 expression in PBMC of HIV-1-positive controllers* versus *progressors*. (a) PBMC of 44 HIV-1 patients (22 controllers (CO) and 22 progressors (PR)) or six healthy donors (HD) were surface stained for human CD3, CD4, CD62L and Tetherin with specific antibodies and analysed by flow cytometry as described in detail in the Material and Methods section. (b) PBMC of 44 HIV-1 patients (22 controllers (CO) and 22 progressors (PR)) or six healthy donors (HD) were fixed, permeabilized, stained for intracellular SAMHD1, p21, RISP and Tetherin and analysed by flow cytometry. (c) In addition, cells were stained for intracellular p24 (here, we included three healthy donors and three HIV-1 patients on antiretroviral therapy (ART)) and (d) CD2 in PBMC and CD4+ T cells. Each data point represents the specific flow cytometry measured relative expression of the indicated cellular factor in one patient. Values were plotted and assessed for statistical significance using the Kruskal–Wallis test with Dunn’s multiple comparison (Graph Pad Prism 6). MFI, mean fluorescence intensity; ns, not significant; * *p* < 0.05; ** *p* < 0.01; *** *p* < 0.001; **** *p* < 0.0001.

RF levels measured by mRNA transcription might not necessarily reflect the total abundance of translated protein. Hence, we quantified RF expression in PBMC of the HIV-1-positive patients by flow cytometry, allowing to assess various parameters in one measurement at a single cell level. PBMC were analysed for cell surface expression of CD3 to identify T cells and further CD4 (Figure 2(a)). In addition, we measured cell surface expression of Tetherin and CD62L, which was recently shown to be specifically downregulated in HIV-1-positive T cells [30]. Another aliquot of the PBMC was permeabilized and stained for intracellular expression of SAMHD1, p21, RISP and Tetherin (Figure 2(b)). Furthermore, we directly assessed the proportion of PBMC positive for the HIV-1 antigen p24 (Figure 2(c)) and included CD2 (Figure 2(d)) in the intracellular stain, as it is a potential marker for latent HIV-1 infection [31]. Importantly, our rational to measure intracellular (i.e. total) CD2 was based on the data of Iglesias-Ussel and colleagues that identified CD2 as a latency marker by mRNA profiling [31]. As a control, RF expression levels and p24 infection rates were also measured in PBMC of healthy donors (HD) and we checked for p24 levels in PBMC of HIV-1 patients on antiretroviral therapy (ART) (Figure 2(c)).

Cell surface levels of CD3, CD62L and Tetherin did not differ between PBMC of viraemic HIV-1 controllers versus progressors, although CD3 and Tetherin levels were generally higher in HIV-1-positive individuals, than in healthy donors (Figure 2(a)). As expected, given that our cohort definitions are valid [32], CD4 levels of PBMC isolated from HIV-1-positive patients were reduced and progressing individuals had lower CD4 levels than viraemic controllers (Figure 2(a)). The differences in CD4 levels are most likely not due to active downregulation in the bulk culture of PBMC but reflect, as expected, the reduced frequency of CD4+ T cells in HIV-1-positive individuals.

Intracellular expression levels of all RFs tested (SAMHD1, p21, RISP, Tetherin) did not reveal any differences in total PBMC potentially associated with HIV-1 control or a progressing course of infection (Figure 2(b)). RFs seem to be induced in HIV-1-positive individuals irrespective of their disease status (Figure 2(b)), which might reflect a generally increased state of immune activation in HIV-1 patients. The percentage of PBMC staining positive for HIV-1 p24 varied strongly between 0.13% and 6.65%, indicating that in some patients a substantial proportion of PBMC might be productively infected (Figure 2(c)).

The p24 antibody clone KC57 is widely used for detection of HIV-1 infection, but was recently also reported to produce some background signal in PBMC [33]. As control, we hence infected PBMC from three healthy donors *ex vivo* and stained for intracellular p24 (Figure S1). We detected infection rates between 16% and 36% with low background staining in mock infected samples in the range of 0.01–0.03%. Background levels in healthy donor PBMC and HIV-1 patients on ART that were treated exactly as the patient PBMC (i.e. no cultivation and stimulation) showed indeed approximately one magnitude higher background p24 staining in the range of 0.1% (Figure 2(c)). Nevertheless, we consistently measured higher p24 signals in the untreated patient PBMC than in our HD and ART controls, altogether demonstrating specific detection of p24 above background levels with our staining procedure (Figure 2(c)).

In sum, the percentage of p24+ cells in PBMC did not differ between HIV-1 viraemic controllers and progressors (Figure 2(c)) and this was also true for the overall distribution of the potential HIV-1 latency marker CD2 (Figure 2(d)). Hence, in agreement with the mRNA profiling, RF expression levels in total PBMC seem not to be associated with the course of HIV-1 infection.

### Expression of RFs in patient PBMC is associated with CD2

**Figure 3.**
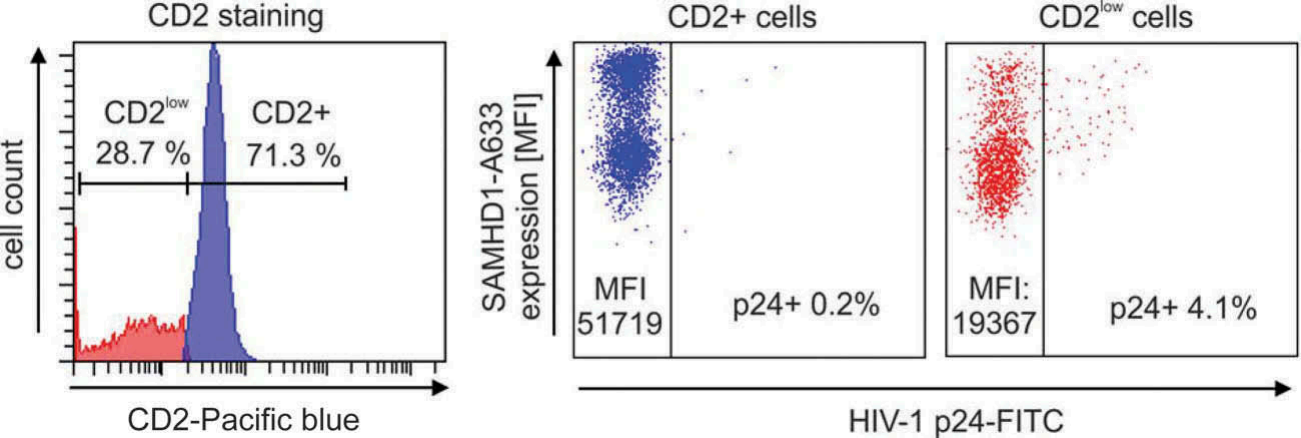
*General gating and exemplary dot plot of SAMHD1 staining in CD2+ and CD2^low^ cells in PBMC of one HIV-1-positive individual (CO14)*. Exemplary primary FACS staining of PBMC of one patient for intracellular CD2, SAMHD1 and p24. We first gated on living cells according to FSC and SSC, then we identified CD2+ vs. CD2^low^ cells by CD2-Pacific blue staining. This allowed to specifically measure RF expression (in the example above SAMHD1 via intracellular Alexa633 stain) and the proportion of HIV-1 p24+ cells (p24-FITC). Indicated are the percentages of cells positively stained for CD2 or p24 respectively, as well as the mean fluorescence intensity (MFI) of SamHD1. See Material and Methods for details concerning the staining procedure.

In the context of our data analyses, we noticed apparent differences in RF expression of CD2+ versus CD2^low^ cells. One example of a representative SAMHD1 staining is presented in Figure 3.

**Figure 4.**
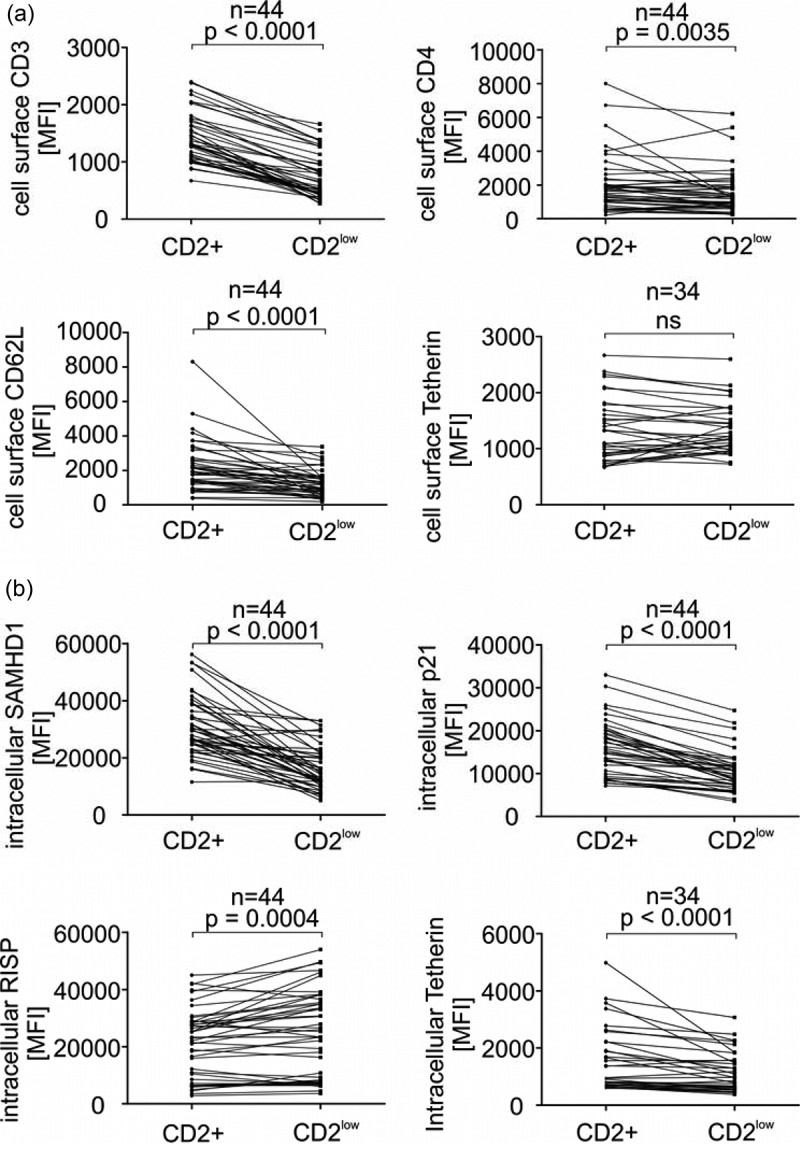
*Surface and intracellular host cell factor expression in PBMC of HIV-1-positive individuals in CD2+* versus *CD2^low^ cells*. (a) PBMC of HIV-1 patients were surface stained for human CD3, CD4, CD62L and Tetherin as well as intracellular CD2 (see Material and Methods) and analysed by flow cytometry. (b) PBMC of HIV-1 patients were fixed, permeabilized and stained for intracellular SAMHD1, p21, RISP and Tetherin as well as CD2 and analysed by flow cytometry. Each data point represents the specific flow cytometry measured relative expression of the indicated cellular factor in one patient. Values obtained from all measurements were grouped according to the CD2 expression (compare Figure 3) and potential differences were assessed for statistical significance with a two-tailed Wilcoxon matched-pairs signed rank test (Graph Pad Prism 6). Respective *p*-values are indicated. N gives the number of analysed patient samples. MFI, mean fluorescence intensity; ns, not significant.

From this patient (CO14), 71.3% of PBMC were highly positive for CD2 (CD2+), whereas the remaining 28.7% of cells expressed considerably lower amounts of CD2 (defined as CD2^low^). The CD2+ cells expressed ~2.5-fold higher levels of SAMHD1 (mean fluorescence intensity [MFI] = 51,719) than the CD2^low^ PBMC (MFI = 19,367). In addition, 4.1% of PBMC in the CD2^low^ fraction were HIV-1 p24 positive in comparison to 0.2% of CD2+ cells (see Figure 3). This prompted us to investigate potential differences in RF expression between CD2+ and CD2^low^ cells across all patients included in our cohort (Figure 4).

We observed markedly reduced cell surface levels of CD3 and CD62L in the CD2^low^ fraction of PBMC. Differences in CD4 were only minor and surface Tetherin levels were similar between CD2+ and CD2^low^ PBMC from all patients (Figure 4(a)). In contrast, intracellular RF levels of SAMHD1 and p21 were strongly reduced in the CD2^low^ PBMC subpopulation as compared to its CD2+ counterparts and we observed the similar albeit less pronounced phenotype for Tetherin (Figure 4(b)). Notably, the putative Rev inhibiting factor RISP, an HIV-1 RF that is specifically active in astrocytes, was slightly upregulated in CD2^low^ PBMC. In a small set of patients, we also measured expression of the recently discovered inhibitor of HIV-1 infectivity SerinC5, which is counteracted by the viral Nef protein [22,23,34] and SerinC5 was clearly reduced in the CD2^low^ fraction of patient PBMC (Figure S2).

Importantly, CD2^low^ cells do not seem to be more activated than their CD2+ counterparts, since *ex vivo* PHA stimulation of PBMC induces CD2 expression (Figure S3). Furthermore, three receptors that are usually upregulated in activated T cells, CD132, CD134 and CD229 [35], were similarly expressed in CD2+ vs. CD2^low^ cells of patients or reduced in the CD2^low^ fraction (Figure S4). In summary, we conclude that the CD2^low^ population of PBMC expresses lower levels of HIV-1 RFs, more specifically SAMHD1 and p21 which are suppressors of HIV-1 reverse transcription and integration [14,15,17,36,37].

### Patient-derived CD2^low^ T cells stain highly positive for the HIV-1 infection marker p24

**Figure 5.**
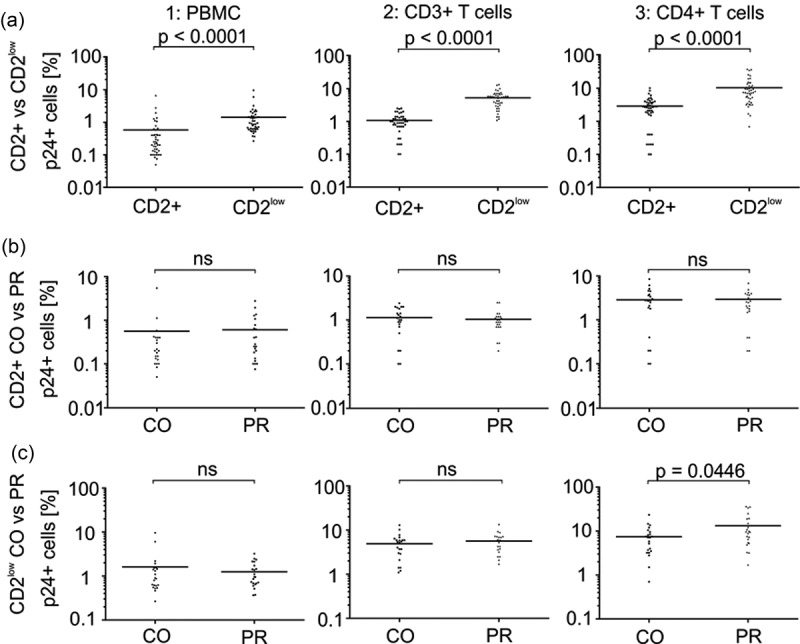
*Levels of HIV-1 p24 antigen in CD2+* versus *CD2^low^ cells of HIV-1-positive individuals*. (a) Intracellular p24 in CD2+ vs. CD2^low^ cells. Intracellular p24 in (b) CD2+ cells from HIV-1 controllers versus progressors and (c) in CD2^low^ cells of HIV-1 controllers versus progressors. The values in a–c were further specifically analysed for CD2 and p24 expression in the whole PBMC population (panel 1), the CD3+ fraction (panel 2) and the CD4+ fraction of cells (panel 3). Each data point represents the specific flow cytometry measured percentage of p24+ cells in the CD2+ or CD2^low^ population. Values were plotted and assessed for statistical significance using an unpaired two-tailed Mann–Whitney test assuming non-Gaussian distribution (Graph Pad Prism 6). N = 44 (compare Table 1). Specific *p*-values are indicated above each subpanel. ns, not significant.

Reduced expression of SAMHD1 and p21 in CD2^low^ PBMC of HIV-1-positive patients could render these cells more permissive for infection. In order to test this hypothesis we analysed the proportion of HIV-1 p24+ cells in the CD2+ versus CD2^low^ fraction of patients’ PBMC (Figure 5(a) and Figure S5 showing primary FACS plots of the stainings).

Of note, patients’ PBMC expressing low levels of CD2 were infected more efficiently (mean infection rate 1.43% p24+ cells) than those expressing high amounts of CD2 (mean infection rate 0.59%; Figure 5(a), panel 1). Inclusion of CD3 or CD4 in our FACS measurements allowed us to monitor the overall infection not only in PBMC but also in the CD3+ or CD4+ fraction of cells (Figure 5(a), panels 2 and 3, see also Figure S5 for representative primary FACS plots). Indeed, infection rates were higher in the overall T cell population (CD3+) and most pronounced, as expected, in the CD4+ cells. Furthermore, the difference in infection rates between CD2+ and CD2^low^ T cells remained. On average 1.08% of CD2+ CD3+ cells were HIV-1 p24+, whereas 5.27% of the CD2^low^ CD3+ cells scored positive for HIV-1 p24 (Figure 5(a), panel 2). In the CD2+ CD4+ cell population, 2.24% were p24+ whereas infection rates of the CD2^low^ CD4+ cells were with 10.33% nearly 5-times higher (Figure 5(a), panel 3). Hence, the CD4+ CD2^low^ fraction of cells within a patient can be infected to relatively high levels.

**Figure 6.**
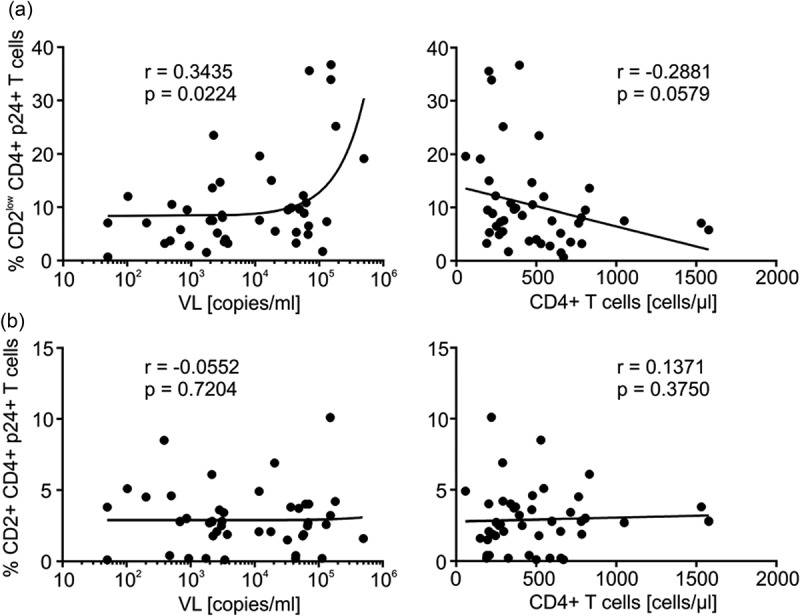
*p24 levels of CD2^low^ CD4+ T cells correlate with HIV-1 viral load in patients*. Levels of p24 in (a) CD2^low^ versus (b) CD2+ CD4+ T cells were correlated to patients’ viral loads (VL) and CD4+ T cell counts (compare Table 1). Regression analyses were done with Graph Pad Prism 6. Indicated are the Spearman rank correlation coefficients (*r*) and the *p*-values.

CD2^low^ T cells might get infected due to the low abundance of RFs. We therefore hypothesized that the CD2^low^ T cell infection rate could be associated with HIV-1 disease status. To test for this, we first plotted the infection rates of CD2+ PBMC (Figure 5(b), panel 1), CD2+ CD3+ T cells (Figure 5(b), panel 2) and CD2+ CD4+ cells (Figure 5(b), panel 3) of HIV-1 patients controlling the infection or showing a progressive course of infection. As expected, CD2+ T cell infection did not differ between both groups. Furthermore, infection rates of CD2^low^ PBMC (Figure 5(c), panel 1) and CD2^low^ CD3+ T cells (Figure 5(c), panel 2) were similar. In contrast, CD2^l^°^w^ CD4+ cells of HIV-1 patients with a progressive course of infection had higher p24 levels than CD2^low^ CD4+ cells isolated from controllers (Figure 5(c), panel 3). More importantly, we measured a direct correlation between p24 levels of the CD2^low^ CD4+ cell fraction and patients’ viral loads (Figure 6(a)) and a strong trend (*p* = 0.0579) towards an inverse correlation with patients' CD4+ T cell counts.

As control, there was no correlation when we plotted the clinical parameters against the fraction of infected CD2+ CD4+ cells (Figure 6(b)). We conclude from these data, that cells with reduced CD2 expression are preferentially HIV-1-infected in patients and p24 levels of CD2^low^ CD4+ T cells are associated with HIV-1 viral loads.

## Discussion

*In vitro* RFs suppress HIV-1 replication at various stages. However, there is sparse and conflicting evidence supporting a relevant role of RFs for lentiviral control *in vivo* [4,5,7,10–13,38]. It is hence unclear if RFs have the potential to lead to new therapeutic avenues. HIV-1 has evolved potent measures to counteract diverse RFs, e.g. Vpu counteracts Tetherin [20,21], Vif inactivates APOBEC3G [39] and Nef antagonizes SERINC3/C5 [22,23,34]. Furthermore, viruses rapidly adapt and evade the host’s specific antiviral immune defence [40]. IFNα induces RFs [1] which serum levels inversely correlate with T cell counts and are positively associated with viral loads and high levels of immune activation predictive for HIV-1 progression [5,6,41]. Therefore, RFs seem not to have a major impact on HIV-1 titres or pathogenesis *in vivo*.

The largest body of evidence supporting an important role of RFs for HIV-1 control *in vivo* stems from studies, in which HIV-1/HCV co-infected individuals were treated with IFNα. In one trial enrolling 860 HIV-1/HCV patients that had not received IFNα treatment before (~80% received ART against HIV-1), IFNα resulted in decreased CD4+ T cell numbers and 0.7 log reduced viral loads [42]. In a small number (*n* = 10) of HIV-1/HCV co-infected patients treated with or without IFNα a slight reduction in cell-associated viral RNA but no effects on plasma viral load, CD4+ T cells or other HIV-1 disease markers was observed [43]. In ART-naïve HIV-1/HCV co-infected patients, IFNα treatment also leads to a reduction of HIV-1 viral loads and a significant induction of RFs [41]. Finally, IFNα monotherapy in therapy-naïve HIV-1 patients had no effects on CD4+ T cell counts but resulted in an ~0.6 log decrease in viral loads, although these effects were highly variable, transient and the patient cohort was small (*n* = 11) [44]. IFNα induces pleiotropic effects and its administration could enhance as well as suppress HIV-1 by various independent mechanisms. Hence, it is difficult to devise definite conclusions on the *in vivo* relevance of RFs for HIV-1 control from the aforementioned studies.

Few studies investigated the expression profile of different RFs in HIV-1-positive individuals controlling the infection without therapy and found either no [7], a subset-specific association [45] or correlation of RF expression with cellular activation status [5]. The latter study, which analysed 42 HIV-1 RFs by mRNA profiling, found enhanced expression of Schlafen11 in CD4+ T cells from elite controllers, whereas there were no differences between controllers and uninfected individuals.

Herein, we comprehensively profiled RF transcriptional levels and relative protein expression in a cohort of therapy-naïve HIV-1 controllers as well as individuals progressing to disease. By this, we challenged the hypothesis that levels of RFs investigated here (SAMHD1, p21, RISP, Tetherin) could influence the pathogenic course of HIV-1 infection and might contribute to HIV-1 control *in vivo*. We report no differences in RF expression in total PBMC of HIV-1 controllers versus progressors (Figures 1 and 2), but CD4 levels were lower in progressors as expected [32], independently confirming the validity of our cohort analyses.

Interestingly, when we included the proposed HIV-1 latency marker CD2 [31], there was a clear and robust association of high HIV-1 RF expression levels with the expression of CD2 across all patients (Figure 4). In line, HIV-1 p24 was increased in PBMC, CD3+ and CD4+ cells with low CD2 levels (Figure 5). From this data, it is tempting to postulate an overall important role of the analysed RFs for cell-associated HIV-1 p24 levels, i.e. viral gene expression. While such a conclusion would be in line with the biological function of RFs and the growing body of *in vitro* results [1,3], it has to be postulated very cautiously. First, as it is the case for most clinical studies, we report correlations, no causalities. Second, our results could also simply reflect higher activation of HIV-1-positive cells, which would lead to increased p24 and lower RF expression. While such a scenario has to be considered, our experimental evidence and the literature do not support this. Cell surface CD2 is associated with T cell activation and increased post TCR stimulation of primary naïve CD4+ T cells [35,46] and PBMC (Figure S3). Furthermore, we measured additional markers on the surface of CD2+ versus CD2^low^ cells (Figure S4) which remained either unchanged between both groups (CD132 and CD134) or were significantly reduced (CD229) on CD2^low^ cells. CD132 and CD134 are cytokine/chemokine receptors and CD229 is a SLAM family member involved in T cell activation. All these receptors are upregulated upon canonical TCR stimulation of naïve T cells [35], strongly indicating that the low RF expression levels of the CD2^low^ T cell population are not a consequence of cellular activation. We also tried to recapitulate the phenotype in *ex vivo* TCR stimulated and experimentally HIV-1-positive CD4+ T cells from healthy individuals and measured CD2 as well as RFs and p24 levels (data not shown). However unfortunately, CD2 levels rapidly and dramatically changed as soon as PBMC or CD4+ T cells were cultured *ex vivo*, precluding the analyses of a potential association between CD2 expression and infection in cell culture experiments.

We herein detected robust p24 levels in the PBMC population of HIV-1-positive individuals, when using the well-established p24 KC57 clone from Beckman-Coulter. In a recent study, the authors also used this clone to detect p24 in patient samples [33]. In agreement to the results of Baxter and colleagues, we found that this antibody generated some background signal in patient PBMC which was in our hands in the range of ~0.1% of cells. This is quite high given that some of our patient samples had p24 levels of 0.13%, which is close to background. On the other hand, the measured p24 levels in our cohort of therapy naïve controllers and progressors were higher throughout than in our controls, i.e. three healthy donors and three patients on ART (Figure 2(c) and Figure S5). In conclusion, although the KC57 p24 antibody generates some background, the reagent allows to measure a specific p24 signal. In agreement, when we gated on CD3+ or CD4+ cells (Figure 5 and Figure S5), the amount of p24+ cells constantly increased, as expected when specifically deconvoluting for HIV-1 target cells. Furthermore, CD2^low^ CD4+ cells from progressing individuals harboured significantly more p24 than those of controllers and viral loads directly correlated with p24 levels in the CD2^low^ CD4+ T cells (Figure 5(c) panel 3 and Figure 6(a)). This indicates that the extent of CD2^low^ CD4+ T cell infection might be linked to the course of HIV-1 infection and viral loads in patients.

Overall, we identify here in PBMC isolated from therapy naïve HIV-1-positive individuals a positive correlation between CD2 and the expression of selected host RFs, mainly p21 and SAMHD1. This phenotype inversely correlates with cell-associated HIV-1 p24 antigen and supports an important role of RFs *in vivo*.

## Notes

To access the supplementary material to this article please see Supplementary Files under Article Tools online.
